# Human adipose-derived mesenchymal stem cells-derived exosomes encapsulated in pluronic F127 hydrogel promote wound healing and regeneration

**DOI:** 10.1186/s13287-022-02980-3

**Published:** 2022-08-08

**Authors:** Yang Zhou, Xing-Liao Zhang, Shou-Tao Lu, Ning-Yan Zhang, Hai-Jun Zhang, Jing Zhang, Jun Zhang

**Affiliations:** 1 Translational Medical Center for Stem Cell Therapy, Shanghai East Hospital, School of Medicine, Tongji University, Shanghai, 200092 China; 2 Key Laboratory of Spine and Spinal Cord Injury Repair and Regeneration of Ministry of Education, Stem Cell Translational Research Center of Tongji Hospital, School of Life Science and Technology, Tongji University, 389 Xincun Road, Shanghai, 200065 China; 3 Shanghai Institute of Stem Cell Research and Clinical Translation, Shanghai, 200120 China; 4 National United Engineering Laboratory for Biomedical Material Modification Branden Industrial Park, Dezhou, 251100 Shandong China; 5 Tenth People’s Hospital of Tongji University, Shanghai, China

**Keywords:** Wound healing, Adipose-derived mesenchymal stem cells, Exosomes, PF-127 hydrogel

## Abstract

**Background:**

Large area skin trauma has always been a great challenge for both patients and clinicians. Exosomes originating from human adipose-derived mesenchymal stem cells (hADSCs) have been a novel promising cell-free treatment in cutaneous damage repair. Nevertheless, the low retention rate of exosomes post-transplantation in vivo remains a significant challenge in clinical applications. Herein, we purposed to explore the potential clinical application roles of hADSCs-Exos encapsulated in functional PF-127 hydrogel in wound healing.

**Methods:**

hADSCs-Exos were isolated from human hADSCs by ultracentrifugation. An injectable, biocompatible, and thermo-sensitive hydrogel Pluronic F-127 hydrogel was employed to encapsulate allogeneic hADSCs-Exos, and this complex was topically applied to a full-thickness cutaneous wound in mice. On different days post-transplantation, the mice were sacrificed, and the skin tissue was excised for histological and immunohistochemical analysis.

**Results:**

Compared with hADSCs-Exos or PF-127 only, PF-127/hADSCs-Exos complexes enhanced skin wound healing, promoted re-epithelialization, increased expression of Ki67, *α*-SMA, and CD31, facilitated collagen synthesis (Collagen I, Collagen III), up-regulated expression of skin barrier proteins (KRT1, AQP3), and reduced inflammation (IL-6, TNF-*α*, CD68, CD206). By using PF-127/hADSCs-Exos complexes, hADSCs-Exos can be administrated at lower doses frequency while maintaining the same therapeutic effects.

**Conclusion:**

Administration of hADSCs-Exos in PF-127 improves the efficiency of exosome delivery, maintains the bioactivity of hADSCs-Exos, and optimizes the performance of hADSCs-Exos. Thus, this biomaterial-based exosome will be a promising treatment approach for the cutaneous rejuvenation of skin wounds.

## Introduction

The skin constitutes the human body’s largest organ, accounting for about 8% of the total weight [1]. It covers the whole surface area and constitutes the first protective barrier to the external environment. So far, large-area skin trauma, burns, frostbite, and chronic wounds remain significant challenges for skin healing [2, 3]. Cutaneous wound healing is complex, initiated immediately after injury, and includes the multicellular overlapping and coordinated phases of inflammation, angiogenesis, granulation tissue formation, re-epithelialization, fibroproliferation or matrix formation, and remodeling [4, 5]. Unhealable wounds usually fail to follow the typical healing cascade, leading to stagnation in chronic inflammation and causing great pain to patients [6, 7].

The recent advancements in stem cell research have enabled stem cell-centered treatment to serve as a prospective alternative for rejuvenating wounds, such as bone marrow-derived MSCs (BM MSCs), umbilical cord mesenchymal stem cells (UC-MSC), and adipose-derived MSCs (ADSC) [8–12]. In particular, the human adipose-derived mesenchymal stem cells (hADSCs) have exhibited remarkable wound repair ability [13–15]. hADSCs can be harvested repeatedly and easily with a minor invasive procedure and a higher yield of MSCs [16]. hADSCs have a longer lifespan, higher proliferative capacity, shorter ploidy time, and senesce later than BM MSCs, and possess a stronger cell division capacity than BM MSCs, making them ideal for cell-based therapy for chronic and persistent conditions [17, 18].

Recent research evidence has documented that exosomes, 40–150 nm particles secreted by cells, can regulate cell-to-cell communication via the transfer of the molecules they carry, including mRNA, miRNAs, and proteins, to target cells [19]. Many studies have indicated that the therapeutic effects of stem cells are more notably dependent on paracrine pathway signals, particularly exosomes [20, 21]. Currently, it is recognized that exosomes derived from MSCs (MSCs-Exos) have a tissue-repair ability equal to or greater than that of MSCs themselves. Recent studies have also documented that MSCs-Exos potentially enhance wound healing [22, 23]. Our previous study has illustrated that exosomes derived from hADSCs (hADSCs-Exos) can promote cell proliferation, angiogenesis, collagen synthesis, and skin barrier function repair, thereby accelerating cutaneous wound healing and revealing that hADSCs-Exos have a tissue-repair capacity more excellent than that of hADSCs themselves [24].

However, the utility of exosomes in treating wounds still faces some challenges since exosomes get cleared rapidly from the application site and survive in vivo for only a short time [25]. Therefore, the combination of exosomes with biomaterials that extend the retention time of exosomes on the wound surface without affecting their biological activity has become a focus of research to develop exosome-based therapies [26]. Tissue engineering-based strategy demonstrates that the utilization of biomaterial-based scaffolds as stem cell delivery and retention platforms can enhance the therapeutic efficiency of stem cells on wound regeneration [27].

Hydrogels have been regarded as promising biomaterials to deliver drugs/cells for wound therapeutics. Pluronic F-127 (PF-127) is a synthetic and biocompatible hydrogel that has been approved for use in humans by FDA. It has been widely adopted as a scaffold for drug delivery, extracellular vesicles (EVs), and encapsulation of cells in tissue engineering [28]. PF-127, used as a wound-repair hydrogel scaffold, has some features, including injectable, biocompatible, and thermo-sensitive [29]. Recent reports also documented the adoption of hydrogels in delivering exosomes to restore vascularization and enhance wound regeneration [26]. However, the comprehensive assessment of the treatment effects and mechanism of combinations of hADSCs-Exos and PF-127 for wound healing was very rare in reports.

In this study, we performed the topical administration of hADSCs-Exos in combination with PF-127. We explored whether topical application of hADSCs-Exos encapsulated in PF-127 hydrogel could accelerate the process of skin regeneration in the cutaneous wound injury mice model. Our results showed that the hADSCs-Exos/PF-127 combination could promote wound healing and cellular proliferation, enhance angiogenesis and collagen synthesis, and accelerate re-epithelialization by slowly releasing exosomes. Furthermore, topically using the hADSCs-Exos/PF-127 combination offers several advantages, including feasible controlled delivery, sustained release of exosomes, and reduced dosing frequency. It is suggested that the topical application of exosome-hydrogel combination is a simple and non-invasive treatment, potentially considered a high-effective, low-toxicity delivery approach.

## Methods and materials

### hADSCs culture and identification

hADSCs were obtained from the Stem Cell Bank of Shanghai East Hospital and were from three different random donors. hADSCs were processed, purified, and confirmed as described in our previous study [24]. The hADSCs cells were cultured in a complete medium containing α-MEM (41,061,037, Gibco, Grand Island, NY, USA) and 10% UltraGROTM-Advanced (HCPCPLCRL50, Helios Bioscience, USA). Osteogenic, chondrogenic, and lipid induction were performed to characterize the hADSCs cell type.

### hADSCs-Exos extraction and identification

After reaching 80–90% confluence, hADSCs were rinsed in dPBS (SH30028.02, HyClone, South Logan, UT, USA) and inoculated with a freshly prepared culture medium for two days. After that, isolation of exosomes from the hADSCs culture medium by ultracentrifugation. The culture medium of hADSCs was centrifuged at 2000 × g for 30 min at 4℃ to get rid of dead cells along with the debris, and then the supernate was transferred to a new 50 ml centrifuge tube, centrifuged at 10,000 × g for 30 min at 4℃ to get rid of other large vesicles. The supernatant would be collected again, put into a new 32ti rotor(Beckman Coulter, Miami, FL, USA), centrifuged at 100,000 × g for 2 h 15 min at 4℃. We removed the supernate, re-suspended the pellets in dPBS, and kept them at − 80 °C. The BCA protein assay kit (23,225, Thermo Fisher Scientific, Waltham, MA, USA) was employed to determine the protein quantitation of hADSCs exosomes. Conventional transmission electron microscopy coupled with NanoSight and Western blotting were adopted to explore hADSCs-Exos morphology, size, and marker expression (CD9, CD63, and CD81).

### PF-127 hydrogel preparation and hADSCs-Exos encapsulation

PF-127 hydrogel preparation was performed according to the previous report [28, 29]. Briefly, 20%, 25%, and 30% (w/v) Pluronic F-127 powder (P2443, Sigma, St Louis, MO, USA) was slowly dissolved in the precooled dPBS buffer solution by magnetic stirring at 4 ℃ overnight, then filtered with a 0.22 μm filter (SLGPR33RB, Merck Millipore, Billerica, MA, USA), and maintained at 4 °C for use. hADSCs-Exos were encapsulated within PF-127 solution with 100ug, and the mixture was blended and stored at 4 °C.

### Release kinetics of PF-127 hydrogel encapsulated hADSCs-Exos

hADSCs-Exos were labeled with PKH26 using a PKH26 Red Fluorescent Cell Linker Mini Kit (MINI26-1KT, Sigma, St Louis, MO, USA). In vitro, to detect hADSCs-Exos released from the PF-127 hydrogel, we mixed hADSCs-Exos with 25% and 30% PF-127 at 4 ℃, then we put them in the transwell upper compartment placed in a 24-well plate (3422, Corning, Corning, NY, USA) at 37 °C, and put 100 ul dPBS in the lower cells. We took the liquid from the lower chamber at 24 h, 48 h, and 72 h. We used the BCA approach to quantitate protein concentration in the lower chamber and calculate the amount percentage of released exosomes. In vivo experiment, the samples of PKH26-hADSCs-Exos encapsulated in the PF-127 hydrogel were topically administrated onto the wound. The concentration of released PKH26-exosomes was calculated by detecting the PKH26 fluorescence intensity in tissues at 24 h, 48 h, 72 h, and 96 h after treatment.

### Wound healing experiments in an animal model

All procedures were approved by the Animal Research Committee of Tongji University. Specific pathogen-free (SPF) male ICR mice (7 weeks old, weighing 28–35 g) were purchased from Shanghai SLAC Laboratory Animal Co., Ltd. The mice model's 1.5 × 1.5 cm full-thickness wound was created as described in our previous study [24]. The 24 mice were randomized into 4 different groups: (1) Control group: no treatment; (2) PF-127 hydrogel group: 100 µl PF-127 hydrogel (25%); (3) hADSCs-Exos group: 100 µg hADSCs-Exos dissolved in 100 µl PBS; and (4) PF-127/hADSCs-Exos group: 100 µg hADSCs-Exos dissolved in 100 µl PF-127 hydrogel (25%). The hADSCs-Exos were applied topically to the wound three times a day, while the PF-127 hydrogel and PF-127/hADSCs-Exos combination were performed once every three days. Post-operative mice were housed individually. The wound areas were measured on days 0, 1, 4, 7, 10, and 13, and calculated with image analysis software. The wound closure rate was calculated using the Wound healing rate = (W0-Wd)/W0 × 100%. W0 wound area on day 0. Wd wound area on day d post-treatment.The survival along with wound healing conditions for every group was documented.

### Histological analysis, immunohistochemically and immunofluorescence staining

Wound samples were harvested on days 4, 7, 10, and 13 after surgery for immunohistochemistry staining. The worst part of each wound healing condition was selected for analysis. Tissue Sects. (5 µm) were mounted on slides for histological analysis. H&E and Masson’s trichrome staining were adopted to visualize the pathological changes in the rejuvenated tissue and collagen formation at the varied healing time.

For immunohistochemically (IHC) and immunofluorescence (IF) staining, the dewaxed sections were washed in PBS, and the endogenous peroxidase activity was quenched by immersion in 2%(v/v) hydrogen peroxide for 5 min. The antigen retrieval was repaired by incubation with sodium citrate buffer for 30 min. After rinsing in PBS, the sections were sealed with 1.5% goat serum at room temperature for 30 min. They were then incubated with primary antibodies anti-Ki67 (ab92742, Abcam, Cambridge, MA, USA), anti-α-SMA (ab124964, Abcam, Cambridge, MA, USA), anti-CD31 (ab281583, Abcam, Cambridge, MA, USA), anti-Collagen I (ab88147, Abcam, Cambridge, MA, USA), anti- Collagen III (ab216430, Abcam, Cambridge, MA, USA), anti-IL-6 (BA4339, Boster Biological Technology, China), anti-TNF-α (BA0131, Boster Biological Technology, China), anti-CD68 (BA3638, Boster Biological Technology, China), anti-CD206 (A02285-2, Boster Biological Technology, China), anti-Cytokeratin 1 (KRT1) (ab185628, Abcam, Cambridge, MA, USA), anti-AQP3 (ab125219, Abcam, Cambridge, MA, USA) overnight at 4℃, and then inoculated with corresponding secondary antibody (goat anti-rabbit or goat anti-mouse, ZSGB-BIO, China). Immunohistochemical staining was developed using the DAB substrate system (DAKO, Denmark).

Images were acquired using a BX53 microscope (Olympus, Japan) and analyzed with Image-Pro Plus 6.0 software. Three animals per group were analyzed for IHC staining. For each sample, we randomly selected at least five fields for analysis. For Ki67, CD68, and CD206 analyses, data are represented as the percentage of positive cell numbers divided by total cell numbers. For *α*-SMA, the collagenous fiber in Masson, collagen I, collagen III, KRT1, AQP3, TNF-*α*, and IL-6 analyses, data are reported as the mean density (IOD/Area) of positively stained regions. The average number of CD31 positive small vessels was manually counted in five random fields. Two independent observers performed all histological assessments.

### Western blotting

Protein concentration Total protein in exosomes was extracted by RIPA (89,901, Thermo Fisher Scientific, Waltham, MA, USA) and quantified using the BCA protein assay kit (23,225, Pierce, Rockford, IL, USA). Fractionation of proteins was done on the 10% SDS-PAGE gels and was transfer-embedded onto Hybond-P polyvinylidene difluoride (PVDF) membrane (ISEQ00010, Merck Millipore, Billerica, MA, USA). Afterward, membranes were blocked and inoculated overnight with the antibodies at 4 °C. Subsequently, the membranes were rinsed in 1 × TBST (C520009, Sangon Biotech, Shanghai, China) and incubated with a secondary antibody in a 37 °C incubator for two hours. Protein bands were analyzed using Amersham Imager 600 system (GE, USA). The antibodies (all from Abcam, Cambridge, MA, USA) used consisted of anti-CD81 (1:1000, ab109201), anti-CD9 (1:1000, ab236630), anti-Tubllin (1:1000, ab7291), and anti-CD63 (1:1000, ab134045).

### Statistical analysis

Statistical analysis was performed using GraphPad Prism software. In the case of more than two groups of samples, one-way ANOVA was used with one condition, and two-way ANOVA was used with more than two conditions. ANOVA analysis was followed by post hoc Bonferroni’s correction for multiple comparisons. *p* < 0.05 was taken as statistically significant; *p-value < 0.05, **p-value < 0.01, ***p-value < 0.001, ****p-value < 0.0001. Data from at least three individual experiments are listed as mean ± standard deviation (mean ± SD) or mean ± Standard Error of Mean(mean ± SEM).

## Results

### hADSCs-Exos isolation and characterization

**Fig. 1:**
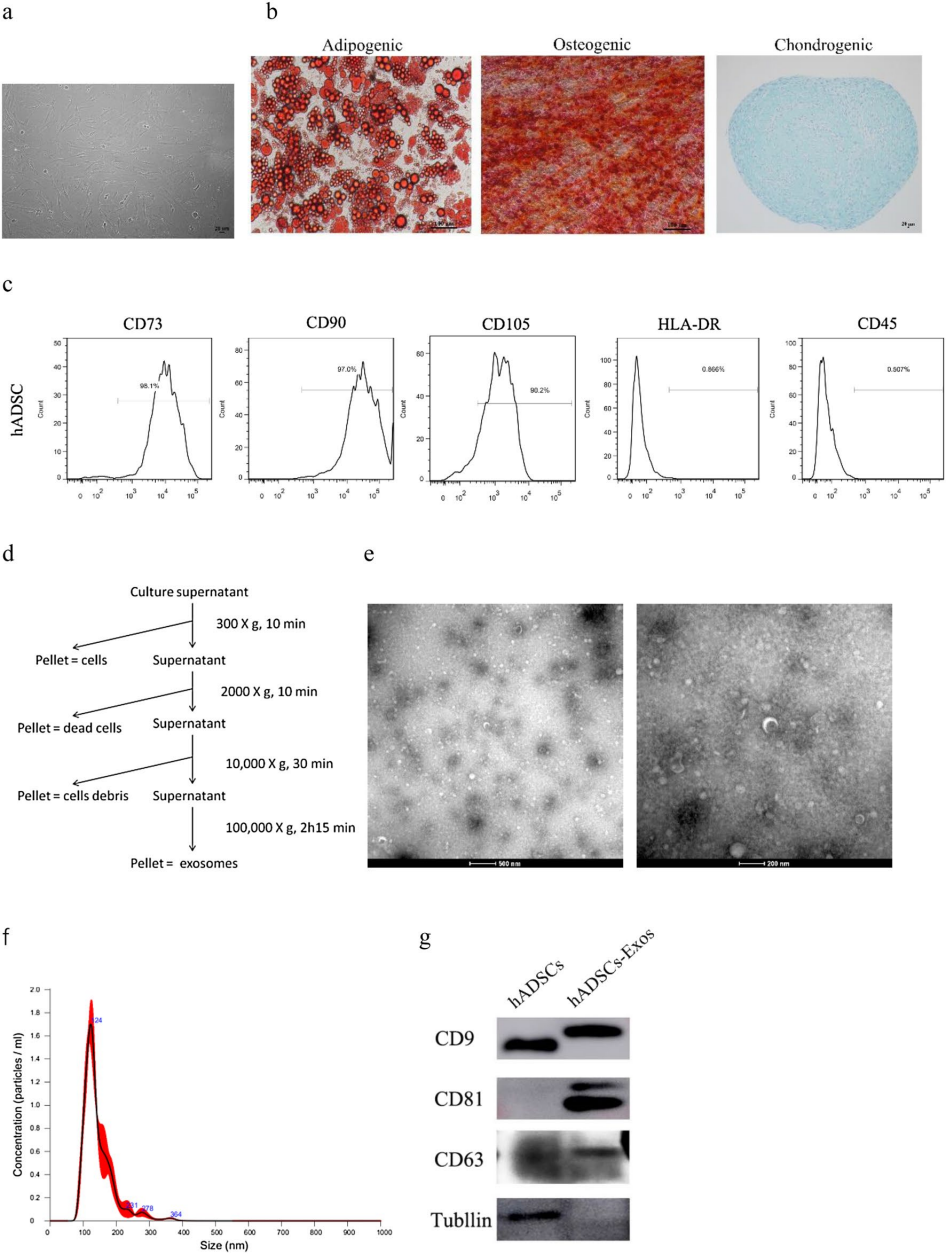
Characterization of hADSCs and hADSCs-Exos. **a** Microscopic images of hADSCs. Scale bar = 20 μm. **b** hADSCs differentiated into adipocytes—Oil Red O (red), osteoblasts – RunX2 (red), and chondrocytes—alcian blue (blue). **c** Detection of the specific markers of hADSCs by flow cytometry illustrated that hADSCs were positive for CD73, CD90, and CD105, negative for HLA-DR and CD45. **d** hADSCs- Exos Ultracentrifugation extraction. hADSCs-Exos were identified by electron microscope (**e**), NanoSight (**f**), and Western blot (**g**)

As illustrated in Fig. 1a, the obtained hADSCs cells displayed classical fibroblast-like morphology and a plastic-adherent property. These cells showed the ability to differentiate into osteoblasts, adipocytes, and chondrocytes (Fig. 1b). Flow cytometry data demonstrated that hADSCs were remarkably positive for MSC surface biomarkers CD73, CD90, and CD105, but negative for HLA-DR and CD45 (Fig. 1c), consistent with the results reported in previous studies. The hADSCs-Exos were harvested from the serum-free medium of hADSCs by ultracentrifugation (Fig. 1d). TEM data exhibited that hADSCs-Exos were saucer-like structurally (Fig. 1e), with diameters between 30 and 100 nm. Nanosight analysis was adopted to assess the diameter size distribution and the particle concentration of hADSCs-Exos (Fig. 1f). Western blot results showed that hADSCs-Exos expressed exosome surface markers, including CD9, CD63, and CD81 (Fig. 1g). All these data illustrated that hADSCs-Exos were successfully isolated in this study.

### Exosome-hydrogel complexes temperature sensitivity and release profile

This study tested the gelling time of 20%, 25%, and 30% (w/v) PF-127 with 100ug hADSCs-Exos at 35 ℃, 36 ℃, and 37 ℃, respectively (Table 1). As the PF-127 concentration increased, the gelling time of PF-127/hADSCs-Exos decreased. The 20% PF-127/hADSCs-Exos composite gelled at 37 °C with 17 s, while the 30% PF-127/hADSCs-Exos composite gelled at 2.7 s at the same temperature. In addition, the gel formation time was inversely proportional to the temperature. The prepared 20% PF-127/hADSCs-Exos mixture was liquid at 4 °C and in a gelation status at 37 °C. The above results revealed that it took more time for 20% PF-127/hADSCs-Exos composite to form gel than the 25% and 30% PF-127/hADSCs-Exos mixtures.

**Table 1:** Gelling Time of Gel Complex in Different PF-127 Concentration and on Different Temperatures (Mean ± sd)

PF-127 concentration (%)	Gelling time at 35 °C	Gelling time at 36 °C	Gelling time at 37 °C
20	20.3 ± 1.5	18 ± 1	17 ± 0.5
25	5.7 ± 1.2	4.7 ± 0.6	3.7 ± 0.6
30	3.7 ± 0.6	3.3 ± 0.6	2.7 ± 0.6

**Fig. 2:**
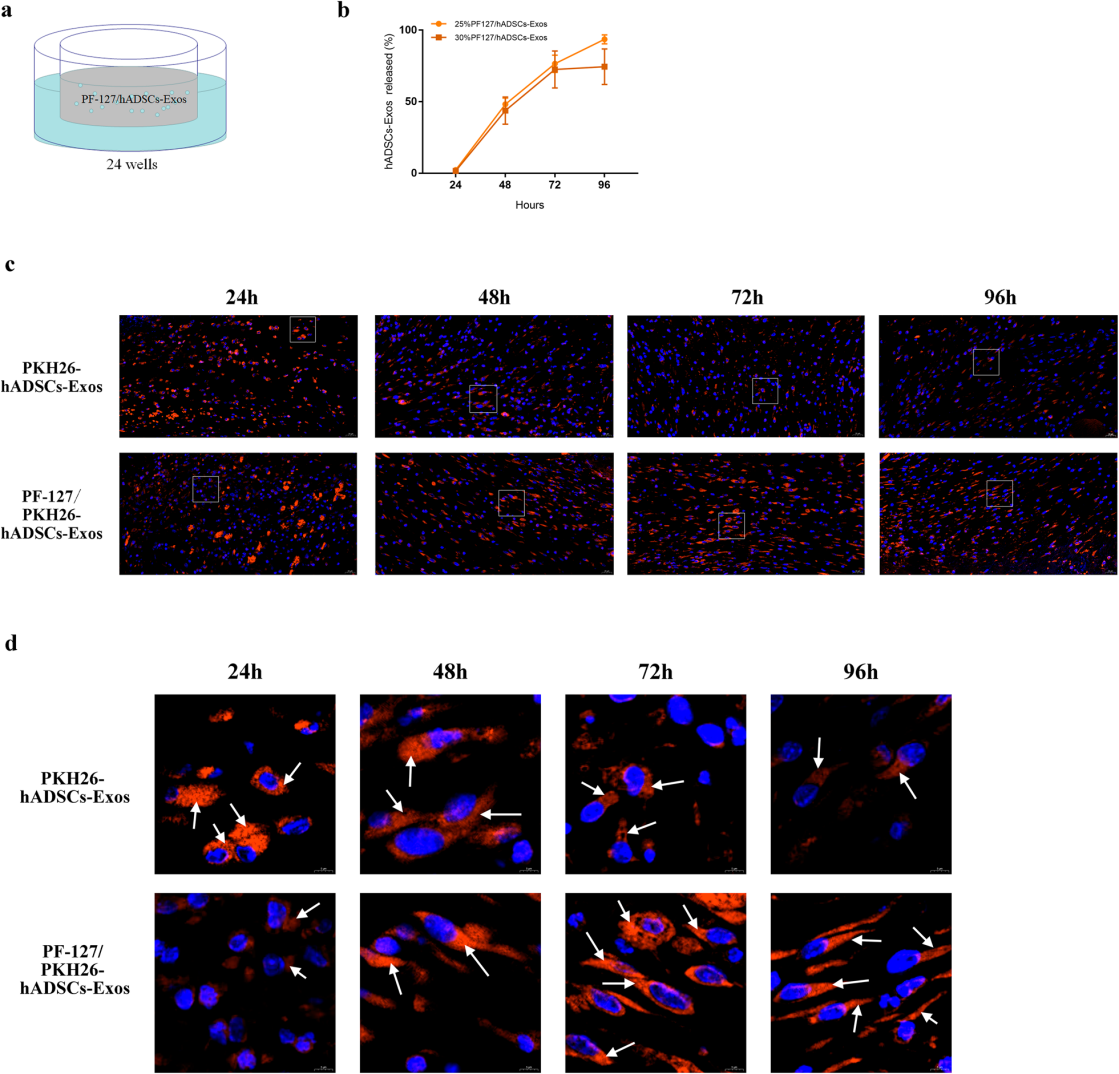
PF-127/hADSCs-Exos complexes allow the stable release of hADSCs-Exos. **a** Schematic representation of the experimental approach. **b** The cumulative release ratio of hADSCs-Exos was detected by protein quantification. **c** PKH26-labeled hADSCs-Exos retention in the site of the wound. Scale bar = 20 μm. d Local magnification of the focal zone in (**c**). PKH26-labeled hADSCs-Exos (red) and DAPI (blue). Arrows indicate hADSCs-Exos. Scale bar = 5 μm

Then, 25% and 30% PF-127/hADSCs-Exos mixtures were chosen to detect the release ability of the hADSCs-Exos. In vitro, the hADSCs-Exos encapsulated in the PF-127 hydrogel could be released steadily over a long time. Compared with the 30% PF-127, 25% PF-127 hydrogel could provide a stable and effective platform for hADSCs-Exos encapsulation and sustained release (Fig. 2a, b). To detect the sustained release of hADSCs-Exos in vivo, hADSCs-Exos were labeled with PKH26 and encapsulated in the 25% PF-127 hydrogel. PKH26 is a lipophilic long-chain carbocyanine dye with highly fluorescent, and it has been applied to the study of EVs and their functions [30]. Thus, we can monitor the localization and migration of the PF-127/ PKH26-hADSCs-Exos to evaluate the contributions of the mixtures in wound healing. After harvesting the wound tissues, the fluorescence microscope analysis revealed an image of the red fluorescence PKH26 dye gathered around the wound 48 h after applying and could still be detected until 96 h, while PKH26 labeled-hADSCs-Exos without PF-127 did not show obvious red fluorescence 48 h later (Fig. 2c, d). These results indicated that 25% PF-127 hydrogel could maintain the sustained release of hADSCs-Exos around the wound area and ensure the effectiveness of hADSCs-Exos for skin wound healing.

### PF-127/hADSCs-Exos promotes cutaneous wound healing in mice

**Fig. 3:**
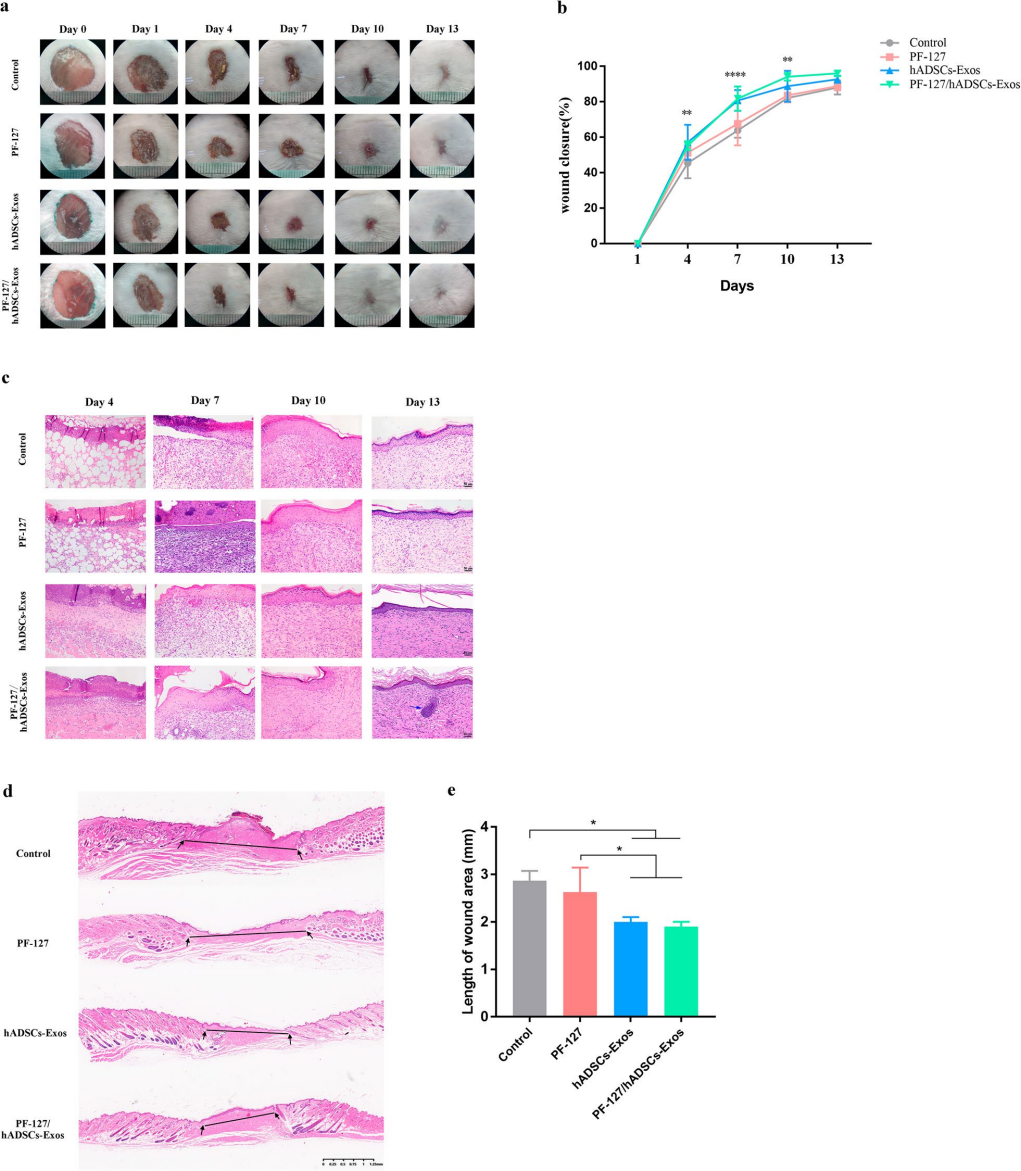
PF-127/hADSCs-Exos complexes promote cutaneous wound healing. **a** Representative images of the skin wound healing in each group on days 0, 1, 4, 7, 10, and 13. **b** Quantitation data of the ratio of the wound healing in each group. **c** Wounded skin sections stained with H&E in diverse groups on days 4, 7, 10, and 13 post-wounding. A representative biopsy from F127/hADSCs-Exo groups showed a normal intact hair follicle (blue arrow). Scale bar = 50 μm. **d** Representative H&E staining images of full-thickness wounds on days 13. Scale bar = 1250 μm. **e** Length between wound edges was calculated. In **b** and **e**, data are shown as mean ± SEM; *n* = 6 for each group. *p-value < 0.05, **p-value < 0.01, ***p-value < 0.001, ****p-value < 0.0001 vs vehicle control group

Full-thickness skin wounds on the backs of mice were created and treated with PF-127/hADSCs-Exos, hADSCs-Exos, PF-127 hydrogel, or no treatment. Representative images of the wound area in each group at 0, 1, 4, 7, 10, and 13 days after surgery are illustrated (Fig. 3a). On days 1, 4, 7, and 10 days after transplantation, the wound-healing rate with the PF-127/hADSCs-Exos composite treatment was remarkably higher than in other groups (Fig. 3b). The histologic structures of the regenerated dermis were analyzed on days 1, 4, 7, 10, and 13. As illustrated in Fig. 3c, the new granulation tissue's epidermis is integrated and thick in PF-127/hADSCs-Exos and hADSCs-Exos groups. However, in the PF-127/hADSCs-Exos group, generation of new hair follicles was evident at the wound center, and proliferating fibroblasts were detected under the epidermis, with orderly and sufficient collagen deposition observed, which were not seen in the hADSCs-Exos alone group. For PF-127 alone and the control groups, there was no evidence of integration in the epidermal structure, with only a thin dermal layer observed. In addition, apparent inflammatory cell infiltration was observed on day 13. Furthermore, we observed a shorter length of wound area under the hADSCs-Exos and PF-127/hADSCs-Exos treatment in contrast with the control wounds (Fig. 3d, e). All these data demonstrated that the PF-127 hydrogel supported hADSCs-Exos survival and biological activity and accelerated the healing process of the wound in mice.

### PF-127/hADSCs-Exos improve cell proliferation and angiogenesis

**Fig. 4:**
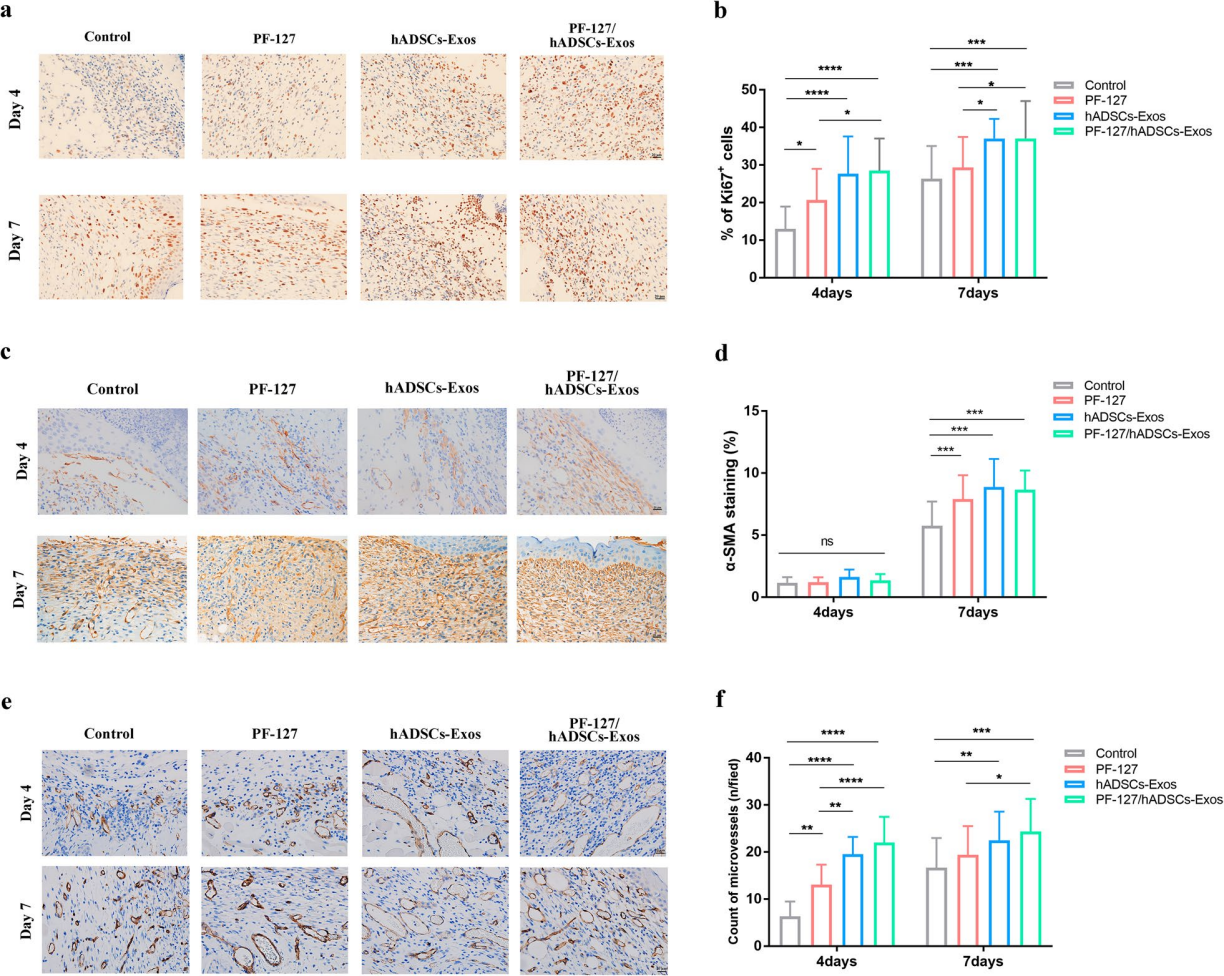
PF-127/hADSCs-Exos improves cell proliferation and angiogenesis. **a** Images illustrate Ki67 immunohistochemistry staining on days 4 and 7. Scale bar = 20 μm. **b** Quantification of the number of Ki67 positive cells in the wound area. **c** α-SMA staining of myofibroblasts in wound bed days 4 and 7 post-operative. Scale bar = 20 µm. **d** Quantification of α-SMA stained tissues. **e** Representative images of IHC of CD31 on days 4 and 7 wound sections. Scale bar = 20 µm. **f** Microvessel density analyses of diverse treatment days 4 and 7 (*n* = 6). In **b, d,** and **f**, data are shown as mean ± SEM; *n* = 6 for each group. *p-value < 0.05, **p-value < 0.01, ***p-value < 0.001, ****p-value < 0.0001 vs vehicle control group

To explore the potential therapeutic mechanism of how the topical application of hADSCs-Exos, PF-127 hydrogel and hADSCs-Exos laden on PF-127 hydrogel affects the cells in the granulation tissues, we performed the IHC analysis of Ki67, CD31, and α-SMA. Ki67 was performed to determine total cellular proliferation. As shown in Fig. 4a and b, the number of Ki67 positive cells in the PF-127/hADSCs-Exos combination group and hADSCs-Exos group significantly increased compared to other groups. Following the PF-127/hADSCs-Exos combination and hADSCs-Exos treatment, the higher expression levels of the myofibroblast marker α-SMA were detected compared to in other groups (Fig. 4c and d). Then, the levels of CD31 were measured to assess newly formed vessels in the regenerated tissue. The endothelial cell marker CD31 was also used to confirm tissue vascularization. Figure 4e and f showed that PF-127/hADSCs-Exos and the hADSCs-Exos group had remarkable blood vessel numbers in contrast with the PF-127 alone and the control groups after seven days of treatment.

### PF-127/hADSCs-Exos can promote collagen synthesis, skin barrier repair

**Fig. 5:**
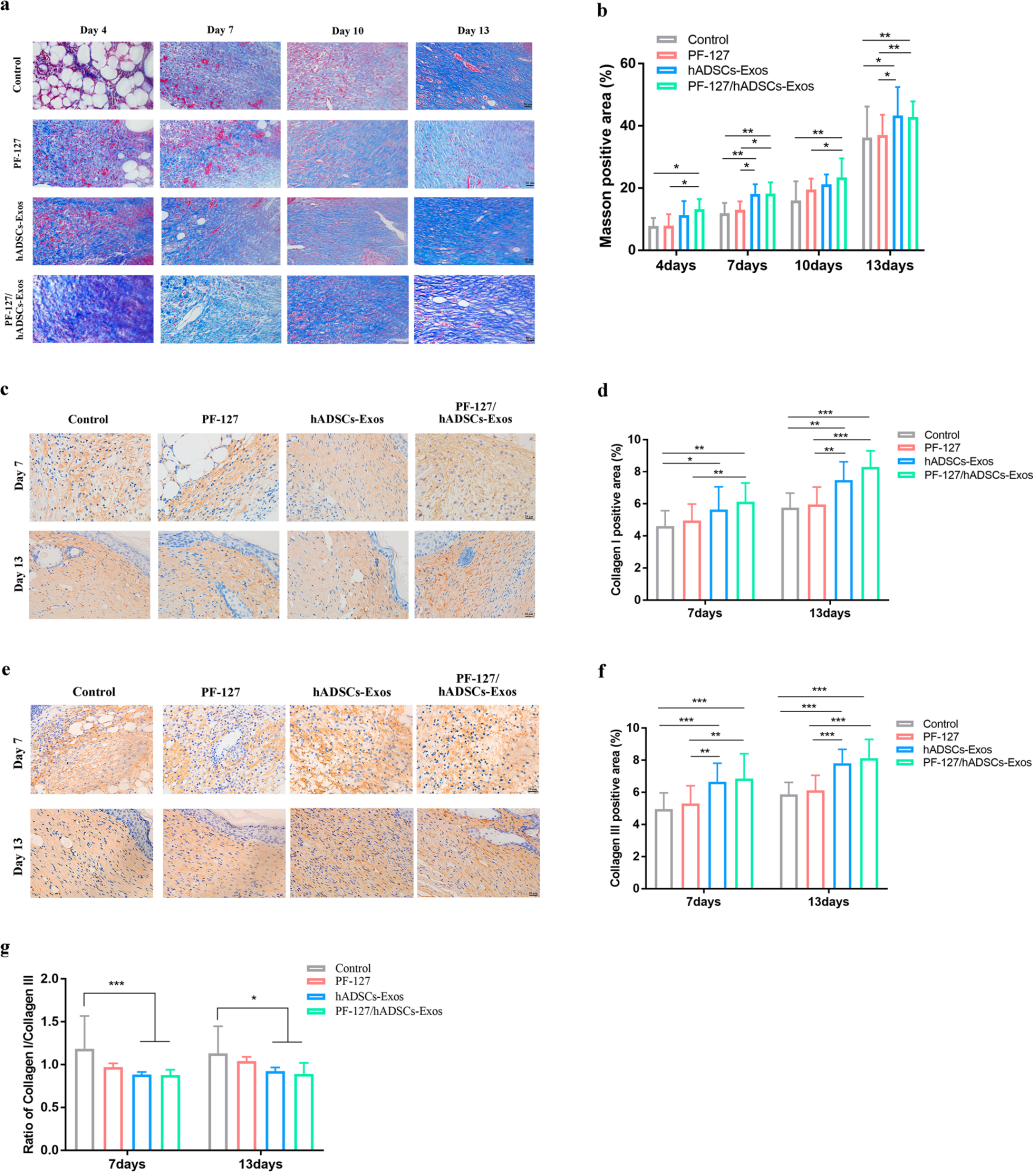
Histochemical analysis of collagen deposition in wounds treated by PF-127/hADSCs-Exos. **a** Masson staining of collagens deposition in different groups on days 4, 7, 10, and 13 post-wounding. Scale bar = 50 µm. **b** Quantification of collagens deposition in different groups. **c** Immunohistochemistry staining images for collagen I at 7, 13 days post-wounding. Scale bar = 20 µm. **d** Relative density analysis of collagen I. **e** Immunohistochemistry staining images for collagen III at 7, 13 days post-wounding. Scale bar = 20 µm. **f** Relative density analysis of collagen III. **g** Relative density analysis of collagen I/collagen III ratios at 7 and 13 days after treatment. In **b, d**, **f,** and **g**, data are shown as mean ± SEM; *n* = 6 for each group. *p-value < 0.05, **p-value < 0.01, ***p-value < 0.001, ****p-value < 0.0001 vs vehicle control group

Proper collagen deposition and remodeling could enhance the tissue tensile strength and lead to a better healing effect. There was remarkably more deposition of newly formed collagen in wounds under PF-127/hADSCs-Exos treatment than in others on days 4, 7, 10, and 13 (Fig. 5a and b). Collagen I and III are the main ECM components in the dermis, and their formation has an indispensable role in wound healing. Thus, immunostaining was employed to explore the collagen I/III contents in the wound tissues. As shown in Figs. 5c–f, the deposition amount of collagen I and III depicted similar changing patterns to Masson staining. With the increase in healing time, collagen Iand III depositions increased in all wounds. At the same time, the PF-127/hADSCs-Exos and the hADSCs-Exos group showed significantly higher intensity and a lower anti-scarring ratio of collagen I to collagen III than other groups (Fig. 5g). It is known that scarless healing fetal wounds accumulate more collagen type III than scarring adult wounds, which have a higher percentage of type I collagen deposition [31, 32]. These data indicated that PF-127/hADSCs-Exos could accelerate the collagen deposition of the wound site, decrease the scar formation, and improve the healing quality of wound tissue, with the same effect as hADSCs-Exos.

**Fig. 6:**
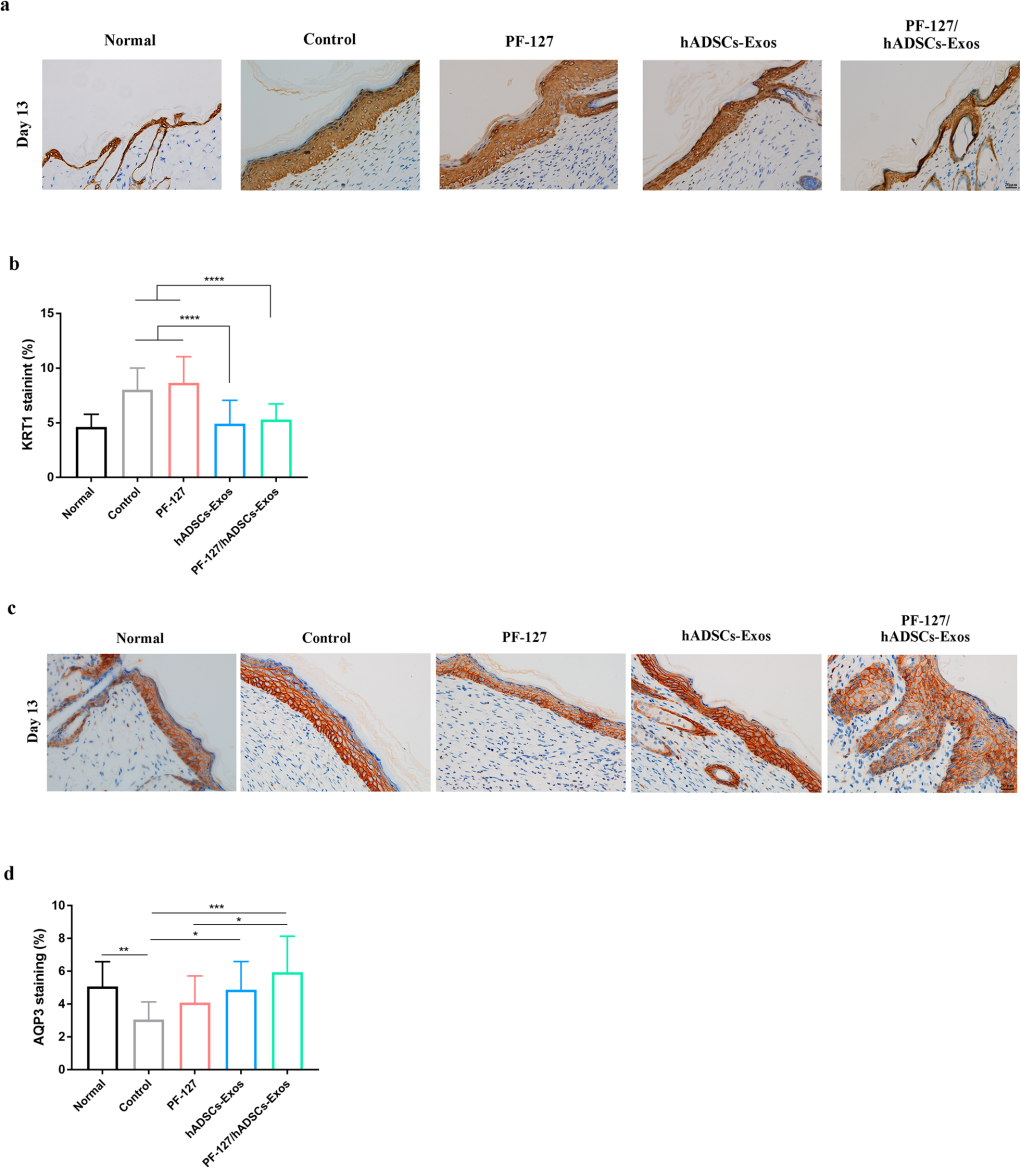
IHC staining of cytokeratin and AQP3 expression in wounds. **a** IHC images of wound sections stained with KRT1 on day 13. Scale bar = 20 µm. **b** Quantification of KRT1 IHC stained tissues. **c** IHC images of wound sections stained with AQP3 on day 13. Scale bar = 20 µm. **d** Quantification of AQP3 IHC stained tissues. In **b** and **d**, data are shown as mean ± SEM; *n* = 6 for each group. *p-value < 0.05, **p-value < 0.01, ***p-value < 0.001 vs vehicle control group

The stratum corneum is the outer layer of the epithelium, which is responsible for the skin barrier permeability and the cornified epithelium resilience. As a remarkable cellular cytoskeleton constituent, keratins constitute the largest subgroup of intermediate filament proteins. KRT1, a keratin family member of cytoskeleton proteins, is primarily expressed in the skin epithelium. On the 13th day, PF-127/hADSCs-Exos and hADSCs-Exos treated wounds exhibited lower expression of KRT1 than other groups (Fig. 6a and b). AQP3 is the dominant aquaporin in human skin, located in the basal layer of the epidermis and the stratum corneum, playing a central role in skin hydration. As illustrated in Fig. 6c and d, on the 13 days, the expression of AQP3 in the PF-127/hADSCs-Exos treatment group was remarkably higher than that in other groups, followed by pure exosomes treated wounds. These data showed that PF-127/hADSCs-Exos improves skin hydration, the elasticity of the skin after trauma, and epidermal permeability barrier function.

### PF-127/hADSCs-Exos can decrease inflammation

**Fig. 7:**
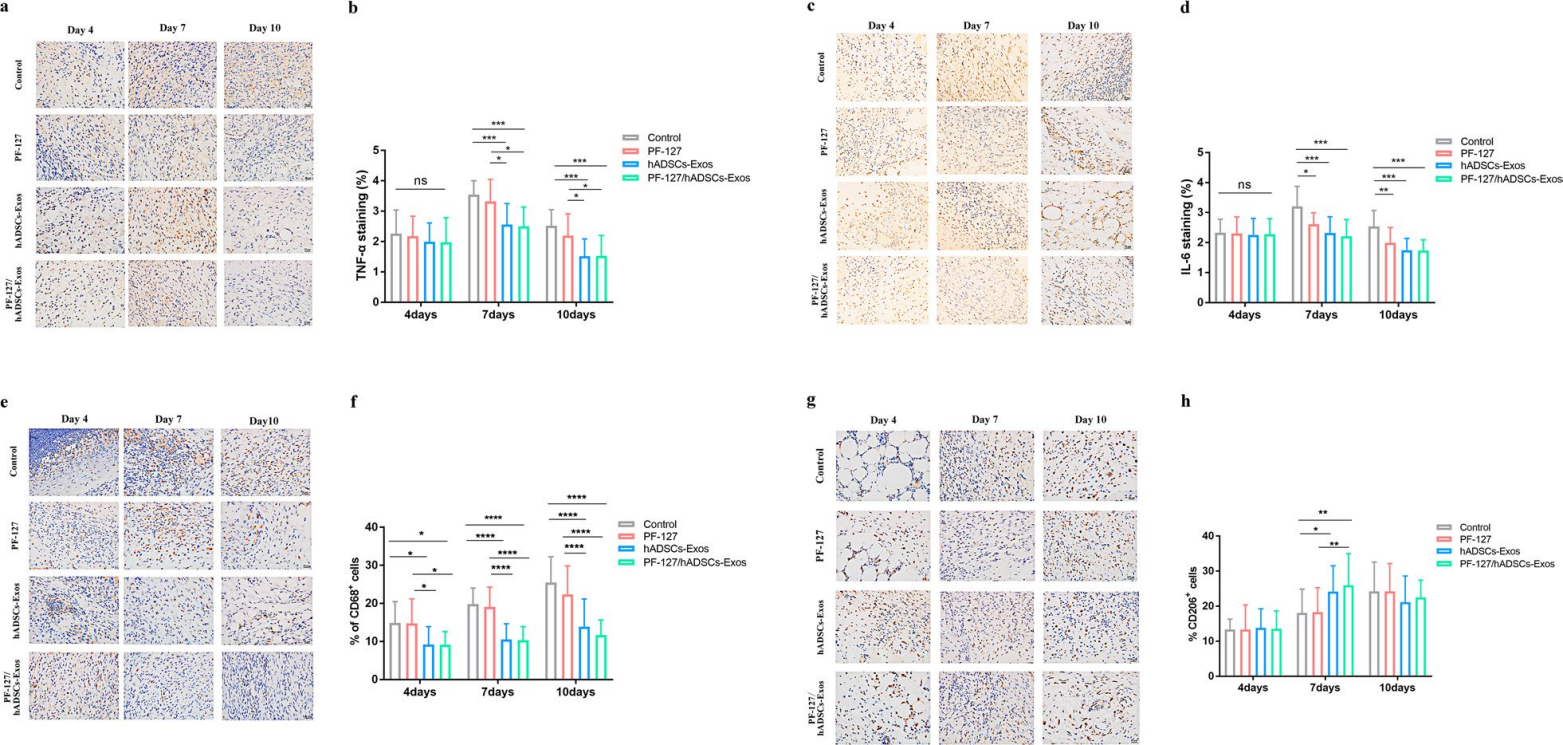
PF-127/hADSCs-Exos complex treatment inhibits inflammatory reaction. **a** Representative images of TNF-α immunostaining at 4, 7, and 10 days after treatment. Scale bar = 20 µm. **b** Quantification of TNF-α^+^ IHC stained tissues. **c** Representative images illustrating IHC results of IL-6 at 4, 7, and 10 days after surgery. Scale bar = 20 µm. **d** Quantification of IL-6^+^ IHC stained tissues. **e** IHC images of wound sections stained with CD68 on days 4, 7, and 10 post-wounding. Scale bar = 20 µm. **f** Quantification of the number of CD68 positive cells in the wound area on days 4, 7, and 10. **g** IHC images of wound sections stained with CD206 at days 4, 7, and 10 post-wounding. Scale bar = 20 µm. **h** Quantification of the number of CD206 positive cells in the wound area on days 4, 7, and 10. In **b, d,** and **f**, data are shown as mean ± SEM; *n* = 6 for each group. *p < 0.05, **p < 0.01, ***p < 0.001, and ****p < 0.0001 versus vehicle control group

Inflammation constitutes the initial response of the phrases of the typical wounding repair. To explore the influence of PF-127/hADSCs-Exos hydrogel treatment on inflammation, the expression level of cytokines, and chemokines, like tumor necrosis factor (TNF)-α and interleukin (IL)-6, were analyzed with IHC. The results showed that PF-127/hADSCs-Exos could alleviate the inflammatory response by downregulating TNF-α and IL-6 (Fig. 7a–d). Besides, macrophages persist across all the stages of the repair process of skin wound healing. M1-like macrophages are expressed in the early inflammatory phase and promote the inflammatory reaction. M2-like macrophages can dampen inflammation and initiate tissue repair by releasing anti-inflammatory cytokines and promoting tissue repair [33]. We employed IHC technology to explore the expression of CD68 (a pan marker for all macrophages) and CD206 (a marker for M2-like macrophages) [34]. The results showed that PF-127/hADSCs-Exos and hADSCs-Exos could decrease the expression level of CD68 and increase the level of CD206 (Fig. 7e–h). These results indicated that PF-127/hADSCs-Exos and hADSCs-Exos could reduce wound inflammation by down-regulating the expression of TNF-α, IL-6, CD68, and up-regulated CD206 expression.

## Discussion

Over the past decade, the number of clinical trials evaluating the efficacy of ADSCs, Stromal Vascular Fraction Cells (SVFs), Human Follicle Stem Cells (HFSCs), Dermal Substitute, and autologous growth factors like Platelet Rich Plasma (PRP) in regenerative plastic surgery has grown exponentially [35]. Wound healing is a complex and dynamic process whereby cells, growth factors (GFs), and the extracellular matrix (ECM) interact to restore the architecture of damaged tissue.

In the past few decades, numerous bi-layer dermal substitutes have been developed and applied for the management of full-thickness skin defects, of which the low-layer porous collagen sponge scaffold (CSS) functions as a dermal regenerate template (DRT) [36–40]. As the extracellular matrix's main protein, collagen scaffolds can provide the physiological 3D structure [41], allowing cellular interaction for migration, proliferation, and differentiation, thus promoting re-epithelialization, revascularization, and closure. However, the complex operation steps of skin graft surgery increase the patient’s pain and prolong the patient’s hospital stay [35, 37, 38]. And the biomaterials often have unique physical characteristics that induce cytotoxicity, extensive scar formation, or uncontrolled inflammation [35, 42, 43].

PRP is another effective wound healing agent. It is a concentrate of platelets and growth factors (GFs) obtained by the centrifugation of venous blood. These GFs and other proteins, such as adhesion molecules and chemokines, interact with the local environment to promote cell differentiation and proliferation, recruiting mesenchymal cells responsible for re-epithelialization and synthesis of angiogenic extracellular matrix [44]. Nevertheless, PRP is limited due to burst release and its short half-life period [45, 46], causing a low concentration of GFs. The combined use of PRP and hyaluronic acid as a bio-functionalized scaffold has shown many advantages for wound healing [47], severe hidradenitis suppurativa [48], and bone exposure [49].

Now, stem cell therapy has emerged as a promising approach for wound healing, hair regrowth, and tissue repair for many years, prevalently represented by ADSCs and human follicle mesenchymal stem cells (HF-MSCs). Among the considerable range of stem cell types examined, ADSCs possess many advantages over other types of stem cells, and their safety and efficacy have been confirmed in wound healing. ADSCs can directly differentiate into other cell types, releasing various growth factors, reducing oxidative stress and apoptosis, promoting angiogenesis, and possessing antibacterial properties to accelerate the process of wound healing [18, 50, 51]. ADSCs and SVFs obtained from the mechanical centrifugation displayed a significantly higher concentration of mature pericytes, ADSCs, and exosomes, when compared to isolated cells with enzymatic digestion [52]. During the last few years, ADSC and SVFs-based cellular therapies have been tested in several clinical settings, like wound healing [51, 53, 54], hair regrowth [55], and breast augmentation [56]. Some studies have highlighted several common points between the biomolecular pathway of ADSCs in wound healing and hair regrowth, particularly the Wnt signaling pathway [57]. More recently, ADSCs even was used as a new regenerative immediate therapy in COVID-19 patient’s treatment thanks to their immunomodulatory and anti-inflammatory activities promoting the wound healing in damaged tissue by a cytokine storm [58, 59]. For soft tissue defects and chronic wounds, ADSCs have been employed in conjunction with a fat grafting technique or with dermal substitute scaffolds and PRP [54, 60]. In recent years, EVs have been considered a new perspective in the therapeutic approach. Stem cell-secreted exosomes and certain biomaterials have been identified as critical components of the wound healing process, and their combination therapy appears to produce significant results.

In this study, we constructed a PF-127/hADSCs-Exos combination and applied it to a mouse cutaneous injury model to efficiently validate its capacity to prolong exosome survival in vitro. The PF-127/hADSCs-Exos composite can significantly promote skin wound healing and maintain the bioactivity of hADSCs-Exos. Our study revealed that PF-127/hADSCs-Exos treatment once three days can still achieve the same therapeutic effect as an hADSCs-Exos treatment three times a day.

hADSCs are regarded as a promising cell arsenal for stem cell treatments and tissue regeneration due to their beneficial features, e.g., easy isolation, self-renewal potential, multi-potency, as well as immune-modulatory effects [17, 61]. hADSCs have been illustrated to accelerate wound healing by promoting neo-angiogenesis and collagen synthesis and reducing inflammation [41, 62]. Our previous studies have shown that topical administration of hADSCs-Exos is more beneficial for promoting skin damage repair than hADSCs themselves [24]. However, the rapid clearance and low survival rate are significant challenges for the topical application of hADSCs-Exos on the wound. The therapeutic efficacy of hADSCs-Exos for wound healing needs to be further improved.

Tissue engineering and regenerative medicine have recently mushroomed as a hotspot approach to facilitating the rejuvenation of wounded tissue [27, 63]. These bioengineering technologies that entail utilizing biomaterial, stem cells, and biologically active factors, have been widely studied in skin soft tissue defects [64]. Numerous reports have documented that PF-127 is a prospective scaffold for encapsulating MSCs or exosomes to promote the regeneration of poorly vascularized tissues, such as epithelial, cartilage, tendons, or even bony and adipose tissues [28]. Hydrogel has unique properties, including thermosensitivity, enabling it to easily encapsulate cells and allowing high cell numbers to adhere at the wound site [65]. Considering that the initial gelation temperature of PF-127 hydrogel reduces with escalating concentration, 25% PF-127 exhibited an average gel time and was thus chosen as the exosome carrier according to our results.

The functionality of exosomes primarily depends on their molecular components consisting of DNA, RNA, and proteins. Nonetheless, MSC-originated exosomes have a short residence time due to their rapid clearance in vivo [25]. To attain a sustained treatment effect, multiple smearing of exosomes is required for topical wound treatment, which is not appropriate for patients. Therefore, increasing retention time using biomaterials to deliver exosomes represents a better strategy. Our results demonstrated that the PF-127 hydrogel could be utilized to ensure a sustained and steady release of hADSCs-Exos in vitro (Fig. 2a, b) and in vivo (Fig. 2c, d).

Wound healing is a delicate and complicated process, and large area skin trauma has always been a great challenge for both patients and clinicians. Skin wound regeneration is complex and entails blood coagulation, inflammation, new tissue formation, and tissue remodeling [2]. Diminished proinflammatory cytokines, compromised neovascularization, and impairment in leukocyte mobilization might disturb and delay wound healing. In this study, we topically applied hADSCs-Exos to the wound sites three times a day and PF-127/hADSCs-Exos once three days. PF-127/hADSCs-Exos group and hADSCs-Exos group exhibited faster healing rates in contrast with those of other groups during the whole healing process, which showed that the PF-127/hADSCs-Exos complex could protect the biological activity of hADSCs-Exos, and release these exosomes continuously, resulting in increased, sustained, as well as rapid wound healing.

To explore the mechanisms of treatment impacts on wound healing at injury sites, we performed the IHC staining of a cellular marker Ki67 to investigate potential mechanisms of how the topical administration of PF-127/hADSCs-Exos, hADSCs-Exos, and PF-127 affects the cells proliferation in the wound tissues. As shown in Fig. 4a and b, more active host cell proliferation (positive Ki67 cells) was observed in the PF-127/hADSCs-Exos and hADSCs-Exos group wounds than in the other groups. Furthermore, the expression levels of myofibroblast marker α-SMA were increased, indicating that both PF-127/hADSCs-Exos and hADSCs-Exos could promote the myofibroblasts formation effectively. The IHC staining of endothelial marker CD31 showed that hADSCs-Exos enhanced angiogenesis in PF-127/hADSCs-Exos and hADSCs-Exos-treated compared with PF-127 alone and control groups. Both PF-127/hADSCs-Exos and hADSCs-Exos could promote cell proliferation and angiogenesis and facilitate the synthesis of granulation tissue at the wound site.

Type I and III collagen is the main component of dermal ECM, which plays an essential role in wound healing. PF-127/hADSCs-Exos and hADSCs-Exos significantly increased the deposition of collagen-I and collagen-III. It has been reported that abundant deposition of collagen-III in the early stage would facilitate healing and result in scarless skin [66–68]. hADSCs-Exos could promote ECM reconstruction in cutaneous wound healing by modulating the ratios of collagen type III to type I by regulating fibroblast differentiation to suppress scar formation [66]. With the increase in healing time, the ratios of collagen I to collagen III were decreased c PF-127/hADSCs-Exos and hADSCs-Exos-treated groups, promoting to form of scar-free wounds.

Meanwhile, PF-127/hADSCs-Exos and hADSCs-Exos regulated the expression levels of KRT1 and AQP3. KRT1 can maintain skin integrity and participates in an inflammatory network in the skin, and its expression level correlates with the degree of inflammation [69]. The elevated AQP3 expression might lead to an increased moisture content of dermal tissues, enhanced skin texture, increased skin elasticity, and the potential to repair wounds and other injuries [70]. In our study, a lower expression level of KRT1 was observed in PF-127/hADSCs-Exos and hADSCs-Exos -treated groups, while a higher expression level of AQP3 was detected.

The four phases of the classical wound repair process begin with inflammation. We found that PF-127/hADSCs-Exos and hADSCs-Exos can alleviate the inflammatory response by downregulating TNF-α, and IL-6, inhibiting M1 macrophage formation and promoting M2 macrophages formation. These data revealed that using the PF-127/hADSCs-Exos complex could maintain the survival of hADSCs-Exos in the inflammatory environment of a wound and retain hADSCs-Exos vitality. Their numbers increase in the inflammation phase, attaining the peak at the granulation tissue formation; however, they decline in the final maturation phase. In our study, the cells with CD68^+^, a pan marker for macrophage, were markedly decreased in the PF-127/hADSCs-Exos and hADSCs-Exos treatment groups, suggesting that the mild inflammatory reactions occurred in the wound area after PF-127/hADSCs-Exos and hADSCs-Exos treated. In addition, the phenotype of macrophages in the wound is influenced by the wound microenvironment and can be roughly divided into M1 and M2 macrophages [33]. M2-like macrophages possess pro-tissue repair as well as anti-inflammatory characteristics. They possess the mannose receptor CD206 [71]. The numbers of CD206^+^ M2-like macrophages were increased in the PF-127/hADSCs-Exos and hADSCs-Exos treatment groups, suggesting that PF-127/hADSCs-Exos and hADSCs-Exos can reduce the inflammatory reaction by promoting the M2-like macrophages formation.

All these above results indicated that more significant numbers of functional hADSCs-Exos were potentially maintained and released continually during PF-127 hydrogel-based delivery to the skin wound, which ultimately contributes to its effectiveness in full-thickness cutaneous wound healing. Nevertheless, the wound repair mechanism induced by hADSCs-Exos is still unclear, and specific signaling pathways need to be explored in our future work.

## Conclusion

In the current study, we successfully applied allogeneic hADSCs-Exos encapsulated in a PF-127 thermosensitive hydrogel for treating wounds. The PF-127/hADSCs-Exos complexes maintained the biological activity of hADSCs-Exos, enhanced cell proliferation, angiogenesis, collagen remodeling, and re-epithelialization at the wound sites, and accelerated the wound healing process. PF-127 thermosensitive hydrogel as a delivery scaffold is suitable for delivering and continuously releasing hADSCs-Exos on the wound site. Compared to the hADSCs-Exos treatment, using the hADSCs-Exos/PF-127 combination topically reduced dosing frequency and optimized the application process for wound treatment. Our study revealed that topically transplanted PF-127/hADSCs-Exos complexes are a promising novel alternative strategy for wound healing.

## Data Availability

Data and reagents will be provided upon availability and reasonable request.

## Abbreviations

hADSCs: Human adipose-derived mesenchymal stem cells
hADSCs-Exos: Human adipose-derived mesenchymal stem cells derived exosomes
TEM: Transmission electron microscopy
NTA: Nanosight tracking analysis
PBS: Phosphate-buffered saline
SEM: Scanning electron microscopy
